# ARID3a expression in human hematopoietic stem cells is associated with distinct gene patterns in aged individuals

**DOI:** 10.1186/s12979-020-00198-6

**Published:** 2020-09-03

**Authors:** Michelle L. Ratliff, Joshua Garton, Judith A. James, Carol F. Webb

**Affiliations:** 1 Department of Medicine, University of Oklahoma Health Sciences Center, Oklahoma City, OK 73104 USA; 2 Department of Microbiology and Immunology, East Carolina University, Greenville, NC 27834 USA; 3 Department of Chemistry and Biochemistry, University of Oklahoma, Norman, OK 73019 USA; 4 Arthritis and Clinical Immunology Program, Oklahoma Medical Research Foundation, Oklahoma City, OK 73104 USA; 5 Department of Pathology, University of Oklahoma Health Sciences Center, Oklahoma City, OK 73104 USA; 6 Department of Microbiology and Immunology, University of Oklahoma Health Sciences Center, Oklahoma City, OK 73104 USA; 7 Department of Cell Biology, University of Oklahoma Health Sciences Center, Oklahoma City, OK 73104 USA

**Keywords:** Immunoaging, Hematopoiesis, ARID3a, Human immunity, Hematopoietic stem cells

## Abstract

**Background:**

Immunologic aging leads to immune dysfunction, significantly reducing the quality of life of the elderly. Aged-related defects in early hematopoiesis result in reduced lymphoid cell development, functionally defective mature immune cells, and poor protective responses to vaccines and pathogens. Despite considerable progress understanding the underlying causes of decreased immunity in the elderly, the mechanisms by which these occur are still poorly understood. The DNA-binding protein ARID3a is expressed in a subset of human hematopoietic progenitors. Inhibition of ARID3a in bulk human cord blood CD34^+^ hematopoietic progenitors led to developmental skewing toward myeloid lineage at the expense of lymphoid lineage cells in vitro. Effects of ARID3a expression in adult-derived hematopoietic stem cells (HSCs) have not been analyzed, nor has ARID3a expression been assessed in relationship to age. We hypothesized that decreases in ARID3a could explain some of the defects observed in aging.

**Results:**

Our data reveal decreased frequencies of ARID3a-expressing peripheral blood HSCs from aged healthy individuals compared with young donor HSCs. Inhibition of ARID3a in young donor-derived HSCs limits B lineage potential, suggesting a role for ARID3a in B lymphopoiesis in bone marrow-derived HSCs. Increasing ARID3a levels of HSCs from aged donors in vitro alters B lineage development and maturation. Finally, single cell analyses of ARID3a-expressing HSCs from young versus aged donors identify a number of differentially expressed genes in aged *ARID3A*-expressing cells versus young *ARID3A*-expressing HSCs, as well as between *ARID3A*-expressing and non-expressing cells in both young and aged donor HSCs.

**Conclusions:**

These data suggest that ARID3a-expressing HSCs from aged individuals differ at both molecular and functional levels compared to ARID3a-expressing HSCs from young individuals.

## Background

The US Census Bureau estimates that nearly a quarter of the US population will be over the age of 65 by the year 2060 [1]. A major consequence of aging is a decline in immune function. Both murine and human studies revealed age-related defects in early hematopoietic development, and functional defects in mature immune cell populations, that result in decreased potentials to mount protective immune responses in aged individuals (reviewed in [2]), as exemplified by increased susceptibility to influenza and pneumonia in the elderly. Human hematopoiesis is a dynamic process requiring complex regulation of multiple gene expression pathways for lineage commitment and resulting in development of diverse numbers of blood cell types [3]. Several studies indicate that aged hematopoietic stem cell (HSC) frequencies are increased in both human and mouse [4, 5]. Bulk CD34-expressing hematopoietic stem and progenitor cells (HSPCs) from aged donors exhibit epigenetic and transcriptional changes that promote self-renewal over differentiation [6, 7]. In old age, cells in the hematopoietic progenitor pool accumulate decreased telomere lengths and DNA damage markers, and their developmental potential becomes increasingly skewed toward myeloid versus lymphoid lineage development [6–9]. Identification of the changes in old age that alter the development of mature immune cells, and possibly contribute to their dysfunction, will require mechanistic studies that better define potential differences in gene regulatory mechanisms critical for lineage choices.

The transcription factor ARID3a is an understudied member of a large family of proteins with epigenetic functions [10–12]. Previous studies indicated that ARID3a can contribute to repression and enhancement of transcription in a cell type-specific fashion [13–15]. Regulation of transcription through ARID3a may be associated with epigenetic functions that affect large subsets of genes and lineage decisions ( [13, 16–18], our unpublished data). ARID3a deficient mice die in utero between days 12 and 14 of gestation, but exhibited a 90% reduction in HSCs numbers in the fetal liver associated with defective erythropoiesis and B lymphopoiesis [19]. Functional loss of ARID3a in B lineage cells, either through ARID3a dominant negative transgenic mice or rare adult ARID3a^−/−^ mice, revealed important roles for ARID3a in B1 B cell lineage development and function [19, 20]. These data were recently confirmed using conditional knockout mice, showing definitively that ARID3a is required for B1 B lineage development in mice [21–23]. Loss of functional ARID3a in B lineage cells in mice directly impaired normal protective immune responses to infection with *S. pneumoniae* [20], an organism associated with pneumonia in aged individuals [24, 25]. Forced expression of ARID3a in mouse B lineage cells resulted in enhanced development of B1 and MZ B cells versus conventional follicular B cells [26], suggesting ARID3a levels can modulate B lineage responses in mice. Mechanisms responsible for generating B1 lineage B cells in man remain controversial [27, 28]. Together, these data identify ARID3a as an important regulator of B lymphopoiesis.

Roles for ARID3a in human hematopoiesis are less clear. We found that ARID3a is variably expressed in healthy human HSPCs, including total CD34^+^ HSPCs, HSCs, multipotent progenitor (MPP), multi-lymphoid progenitors (MLP), and multi-myeloid progenitors (MMP) derived from adult peripheral blood [29], but the functional significance of expression in those progenitors is not clear. In functional studies with human cord blood HSPCs, where ARID3a expression dominates the majority of those cells, manipulation of ARID3a resulted in skewing of lineage development with promotion of myeloid over lymphoid lineage differentiation upon loss of ARID3a expression and increased B lymphopoiesis upon over-expression of ARID3a [30]. ARID3a expression in circulating peripheral blood HSPCs from lupus erythematosus patients is upregulated compared to similar cells from healthy individuals, although the role of ARID3a in those cells is unknown [29]. These data suggest the need for further experiments to determine how ARID3a levels affect adult human hematopoiesis.

We hypothesized that one explanation for reduced B lymphopoiesis and increased numbers of myeloid cells in aged versus young individuals is that ARID3a expression is reduced in HSCs from healthy aged individuals compared to healthy young individuals, or that its function in those cells is impaired. Our results indicate that peripheral blood HSCs from aged donors exhibit reduced frequencies of ARID3a-expressing cells compared with young donors. Furthermore, modulation of ARID3a levels in both aged and young donor-derived HSCs altered B lymphopoiesis in vitro. Finally, single cell RNA-seq analyses revealed unexpected differences in gene expression patterns in *ARID3A*-expressing progenitors from aged versus young individuals.

## Results

**Fig. 1:**
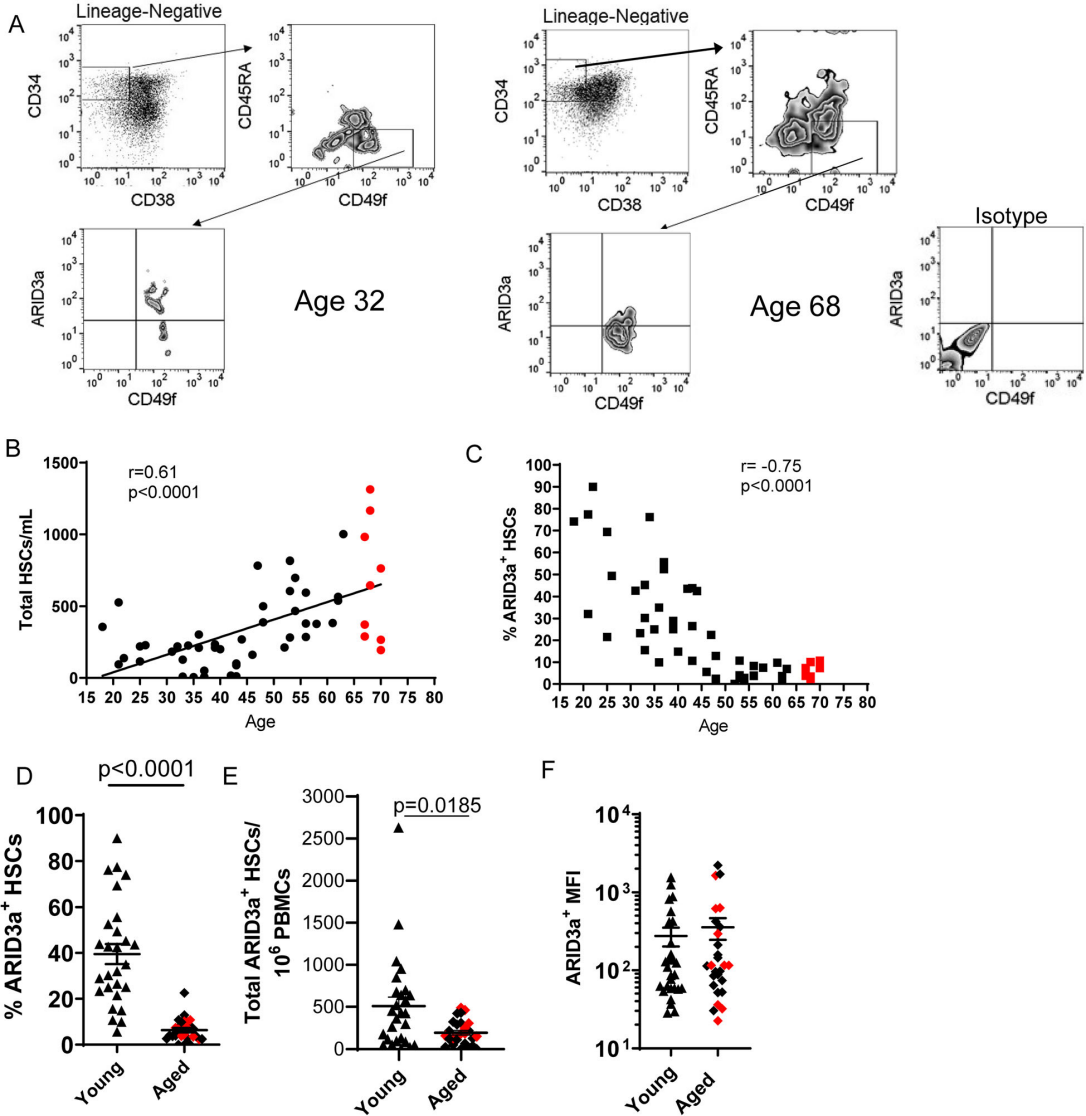
PBMCs from aged individuals show reduced frequencies of ARID3a^+^ HSCs. Human HSCs expressing ARID3a were identified by flow cytometry from total PBMCs of 54 healthy adults (ages 18–70). **a** Representative gating from young (left) and aged (center) donors, with an isotype control for ARID3a (right) are shown. **b** Numbers of HSCs/ml of blood are plotted against age with a line of best fit. Red samples indicate samples from donors ≥65 years of age. **c** Frequencies of ARID3a^+^ HSCs are plotted against age. Frequencies (**d**) and numbers (**e**) of ARID3a^+^ HSCs for young (triangles) and aged (diamonds) donors are shown. **f** Mean Fluorescence Intensities (MFI) of ARID3a^+^ HSCs from young and aged donors are presented. Averages and standard error bars are shown. Statistical significance was determined by Mann Whitney U test and Pearson’s Correlation

We assessed numbers of ARID3a-expressing HSCs (lin^−^CD34^+^CD38^−^CD45RA^−^CD49f^+^) from the peripheral blood of 54 healthy adults of varying ages by flow cytometry (Fig. 1a), as previously described [29, 30]. Total HSC numbers increased with age (*p* < 0.0001) (Fig. 1b), consistent with reports by others that aged individuals (red dots) have increased numbers of HSCs [4–6, 31, 32]. Further analyses of those cells for ARID3a expression, revealed decreases in frequencies of ARID3a^+^ HSCs with increasing donor age (≤10% ARID3a^+^ HSCs), in donors over the age of 50 (Fig. 1c). Based on these data, for our purposes, we defined “young” as under the age of 50 and “aged” as 50 years of age and over but show donors over 65 as red symbols. The frequency of ARID3a^+^ HSCs in aged donors was reduced by an average of 80% compared to young donors (Fig. 1d), but total ARID3a^+^ HSC numbers were not statistically different between young and aged donors (Fig. 1e). Similarly, the amounts of ARID3a within HSCs varied among individuals, but were not statistically different between young and aged individuals (Fig. 1f). Data from individuals over 65 (red symbols) did not differ from individuals over 50 in any of these characteristics. These data show average absolute numbers of ARID3a-expressing HSCs are maintained with age, but that relative frequencies of ARID3a^+^ HSCs in the peripheral blood are decreased with increased chronologic age.

**Fig. 2:**
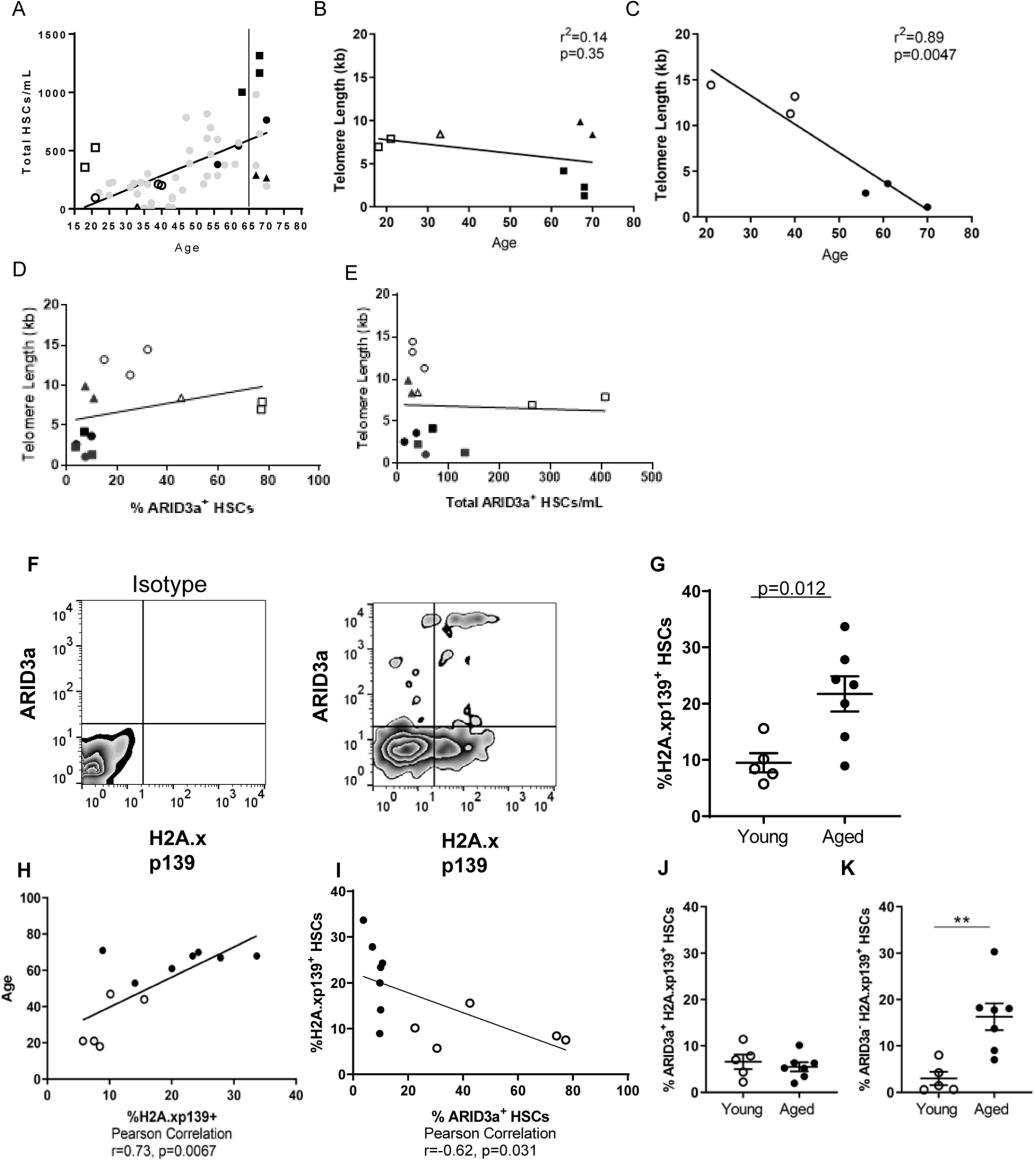
ARID3a expressing aged HSCs are not preferentially associated with markers of biological age. **a** Select HSCs from those presented in Fig. 1b were used to prepare genomic DNA and were assessed for telomere length by qPCR. Solid black points indicate aged samples, while open points indicate young samples. Square denote samples above the line of fit, triangles fall below the line and circles correspond with the line. Telomere lengths of HSCs from samples above and below (**b**), or on/near the trend line (**c**) are plotted against age. Telomere lengths are plotted against frequency (**d**), and total numbers of ARID3a + HSCs (**e**). **f** A representative flow cytometric plot of HSCs analyzed for ARID3a and H2A.xp139 is shown. **g** Frequencies of H2A.x^+^ HSCs from young (*n* = 5) and aged (*n* = 7) donors are shown. **h** Frequencies of H2A.x^+^ HSCs are plotted against age. **i** Frequencies of H2A.x^+^ HSCs are plotted against frequencies of ARID3a^+^ HSCs. Frequencies of H2A^+^ARID3a^+^ HSCs (**j**), and H2A^+^ARID3a^−^ HSCs (**k**) are plotted for young and old donors. Averages and standard error bars are indicated. Statistical significance was determined by Mann Whitney U test and Pearson’s Correlation. **, *p* = 0.0051

Although numbers of HSCs increase with chronologic age (Fig. 1b), there was considerable variability in numbers of HSCs in aged individuals and in some young individuals. We considered that this variability might reflect variations in biological age versus chronological age. Telomere length has been used as a biologic measure of cellular age [33, 34], and telomeres in hematopoietic cells from aged persons have been reported to be shortened compared to those from younger individuals [8]. Therefore, we chose individual HSC samples of both young and aged donor HSCs in Fig. 1a for assessment of telomere lengths (indicated in Fig. 2a). Aged samples with reduced numbers of HSCs (closed triangles) had longer telomeres than those samples with higher numbers of HSCs (closed boxes) (Fig. 2b). The two young individuals with the highest numbers of HSCs (open boxes) showed an average telomere length of 7.4 Kb while HSCs from young individuals with lower HSC numbers (open triangles) had an average of 13 Kb telomere length (Fig. 2b). Others reported an average of 18 Kb for telomere lengths in hematopoietic progenitors from young individuals [8]. Measurements of telomere lengths from other individuals of various ages (circles, Fig. 2c), showed good correlations between telomere length and chronologic age. Neither the frequency of ARID3a^+^ cells (Fig. 2d), nor the total numbers of ARID3a^+^ cells per sample (Fig. 2e) in the HSCs for which we performed telomere analyses were associated with telomere length. Therefore, no correlation was observed between ARID3a status and telomere length.

Another molecular marker associated with aging is accumulation of DNA damage as indicated by accumulation of H2A.xp139 (H2Ax) [9, 35]. This assay allows direct comparison of ARID3a levels with the aging marker via flow cytometric analyses (Fig. 2f). As expected, aged donor HSCs displayed an increased frequency of H2Ax as compared to HSCs from young donors (Fig. 2g). There was also a significant positive correlation between age and H2Ax expression (Fig. 2h), and a negative correlation between frequencies of ARID3a and H2Ax (Fig. 2i). Total frequencies of ARID3a-expressing cells with H2Ax marks associated with DNA damage were not different in HSCs derived from young versus aged individuals (Fig. 2j). However, increased numbers of H2Ax-marked HSCs without ARID3a occurred in aged individuals compared to young individuals (Fig. 2k), suggesting the possibility that increases in total HSCs with age may occur as an expansion of cells that do not express ARID3a. These data suggest that ARID3a^+^ HSCs from aged donors do not reflect cells that have accumulated increases in DNA damage to a greater degree than in young individuals.

**Fig. 3:**
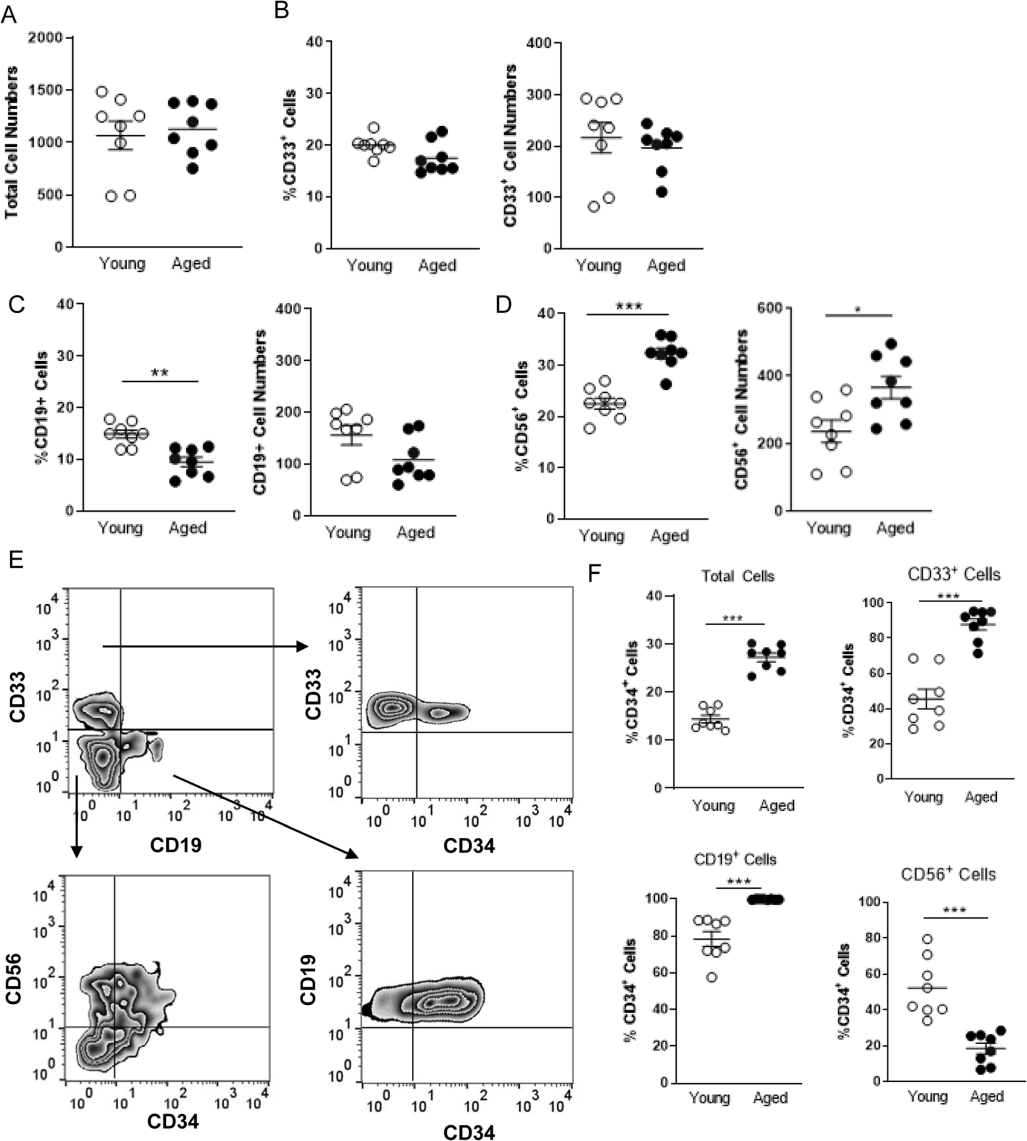
Aged donor HSCs exhibit reduced B lineage development in vitro. Sorted HSCs from young and aged donors (*N* = 8 each) were seeded at 100 cells/ well in triplicate cultures that support multilineage development and were assessed by flow cytometry after 3 weeks. **a** Total numbers of cells from young (open) and aged donor (closed) cultures are shown. Frequencies of CD33^+^ myeloid cells (**b**), CD19^+^ B lineage cells (**c**), and CD56^+^ NK cells (**d**) were evaluated. **e** A representative flow cytometry plot reveals co-expression of CD34 on some cells with lineage markers. **f** Total frequencies of CD34^+^ cells, as well as cells co-expressing mature lineage markers are shown. Means and standard error bars are presented. Statistical significance was determined by Mann Whittney U test; *, *p* = 0.038, **, *p* = 0.0019, ***, *p* < 0.0003

We hypothesized that ARID3a-expressing cells from aged individuals could still be functionally deficient in generating B lymphoid responses that are dependent on ARID3a [20]. Therefore, we explored the developmental potential of aged versus young donor derived HSCs using in vitro cultures that allowed development of B lymphoid, natural killer (NK) and myeloid lineage cells [36]. Because ARID3a is an intracellular protein, it is not possible to isolate those cells via flow cytometry for use in vitro. After 3 weeks in culture, both young and aged donor HSCs gave rise to the same total numbers of cells (Fig. 3a). In these in vitro cultures, total percentages of CD33-expressing myeloid cells were equivalent in cultures initiated with HSCs from young or aged donors (Fig. 3b). HSCs from aged donors generated reduced frequencies of B lineage cells compared to those from young donors (Fig. 3c). Consistent with previous studies [37], aged HSCs generated increased percentages of NK cells compared to young HSCs (Fig. 3d). All HSCs originally express CD34 and lose that surface marker as they differentiate into various hematopoietic lineages [38–40] . Therefore, as a means of investigating maturation of cells in these cultures, we assessed numbers of cells of each lineage that retained CD34 expression (Fig. 3e). Total frequencies of CD34^+^ cells were greatly increased in cultures derived from aged versus young donor HSCs (Fig. 3f). In the aged donor HSCs, the majority of both CD33 and CD19-expressing cells also maintained CD34 expression, compared to lower percentages of monocyte and B lymphoid cells derived from young donor HSCs (Fig. 3f). Conversely, frequencies of CD34-expressing NK lineage cells were reduced in aged HSC donor derived cultures compared to those obtained from young HSC donors (Fig. 3f). These data confirm previous results demonstrating reduced B lineage development from aged donor HSCs [6], and extend those studies to suggest that the B lymphocyte and myeloid lineage cells derived from aged donor HSCs may be less mature than those derived from young donors as reflected by maintenance of CD34 expression.

**Fig. 4:**
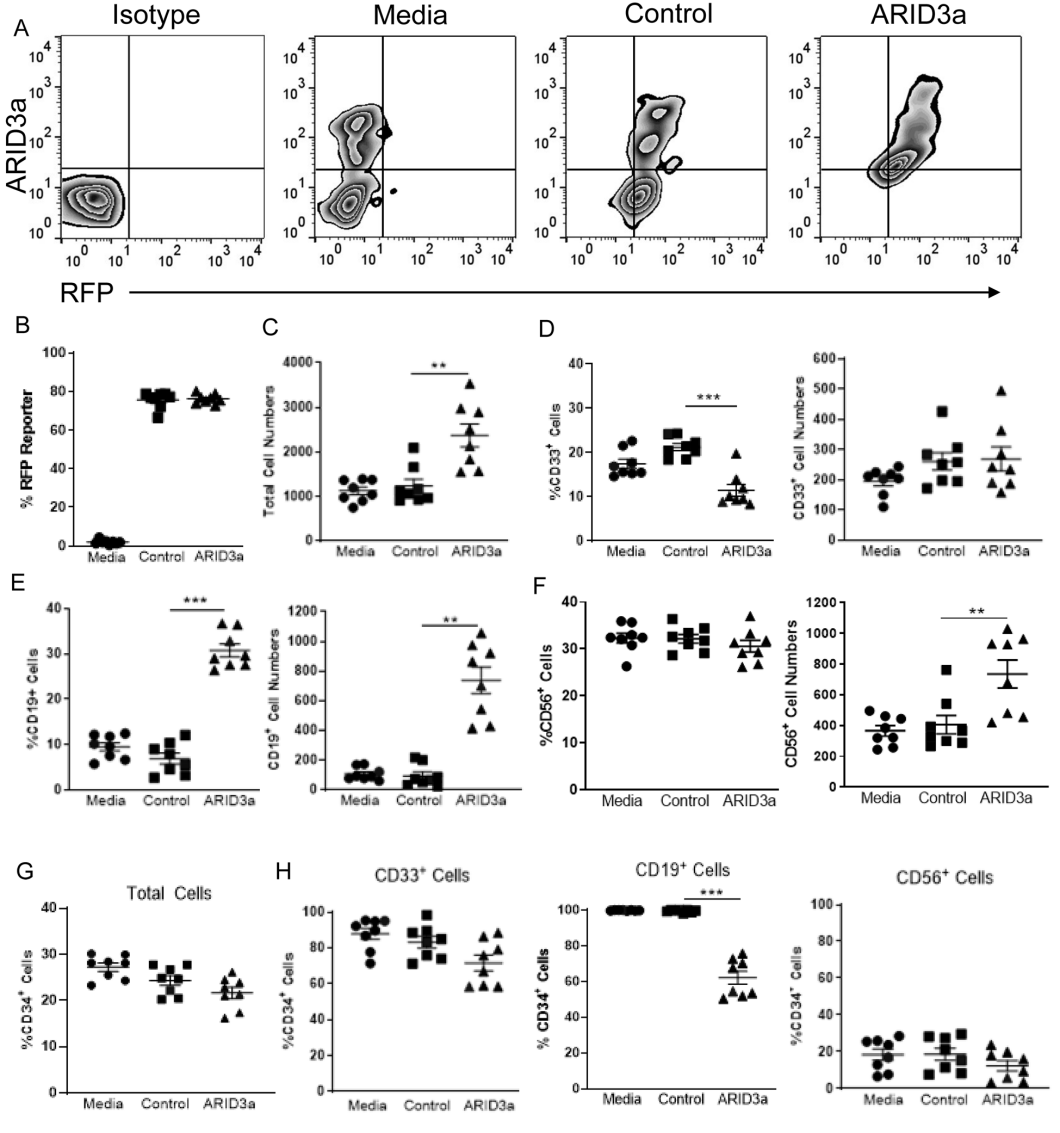
Overexpression of ARID3a in aged donor HSCs increases B lineage cell numbers. The aged donor HSCs from Fig. 3 were untreated (media, circles), or transfected with lentivirus vector controls (control, squares) or lentivirus containing full length ARID3a (ARID3a, triangles) for 24 h, plated as triplicate cultures and analyzed by flow cytometry. **a** Relative transfection efficiencies indicated by co-expression of RFP at the end of the cultures are shown. **b** Total numbers and frequencies of CD33^+^ myeloid cells (**c**), CD19^+^ B lineage cells (**d**), and CD56^+^ NK cells (**e**) are shown. Total frequencies of CD34 expressing cells (**f**) and percentages of lineage-directed cells co-expressing CD34 (**g**) are shown. Means and standard error bars are shown. Statistical significance was determined by Mann Whittney U test; **, *p* < 0.0093, ***, *p* < 0.0003

To determine if modulation of ARID3a levels affected the developmental potential of HSCs, we first forced expression of ARID3a in aged donor HSCs. ARID3a expression was inferred from co-expression of RFP in the transfecting vector, which revealed > 80% RFP-expressing cells (Fig. 4a, b). ARID3a-transfected cultures had increased total cell numbers compared to both untransfected, media only and RFP-only vector controls (Fig. 4c). CD33-expressing myeloid lineage cells were decreased in frequency after ARID3a expression, but total cell numbers remained equivalent (Fig. 4d). As predicted, ARID3a over-expression in aged donor HSCs led to large increases in both B lineage cell frequencies and cell numbers (Fig. 4e), indicating that increased ARID3a expression in adult-derived HSCs from aged donors drove B lymphocyte development in vitro. NK frequencies did not differ among the three culture conditions, but total numbers of NK cells were increased on average in the ARID3a-expressing cultures (Fig. 4f). Examination of CD34 expression in these cultures revealed only slight differences in CD34 expression between ARID3a transfected cultures and the controls except in the CD19^+^ B lineage cells where CD34 expressing cells were significantly reduced in frequency compared to the controls (Fig. 4g and h). These data reveal that increased ARID3a expression in aged donor HSCs generate increased numbers of mature B lineage cells.

**Fig. 5:**
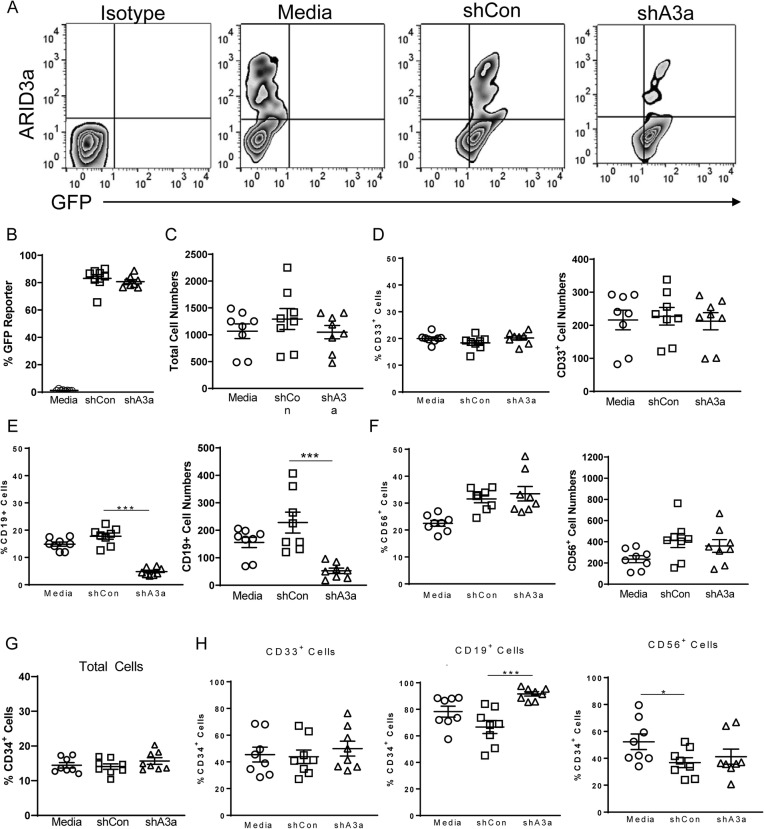
Inhibition of ARID3a in young donor HSCs reduces B linage cell numbers. Young donor HSCs from Fig. 3 were untreated (media, circles), or transfected with lentivirus containing a scramble control shRNA (shCon, squares) or an ARID3a-specific shRNA (shA3a, triangles), and were cultured and assessed as in Fig. 4. **a** Relative transfection efficiencies at the end of the culture are indicated by GFP-co—expression. Total cell numbers (**b**) and frequencies of CD33^+^ myeloid cells (**c**), CD19^+^ B lineage cells (**d**), and CD56^+^ NK cells (**e**) are shown. Total frequencies of CD34^+^(**f**) and CD34-co-expressing lineage-directed cells are shown. Means and standard error bars are presented. Statistical significance was determined by Mann Whittney U test; *, *p* < 0.04, ***, *p* < 0.0003

Similarly, inhibition of ARID3a expression in young donor HSCs using shRNA vectors that co-expressed GFP revealed > 80% GFP-expressing cells, suggesting that ARID3a was reduced in those cells as well (Fig. 5a, b). No differences were observed in total cell numbers between controls and ARID3a-inhibited cultures were observed (Fig. 5c), nor were differences observed in CD33-expressing monocyte or NK lineage cells between vector control and ARID3a-inhibited cultures (Fig. 5d and f). However, frequencies and total cell numbers of B lineage cells without ARID3a were dramatically reduced compared with media and control shRNA cultures (Fig. 5e). While total frequencies of CD34-expressing cells did not differ appreciably among the three culture conditions (Fig. 5g), the majority of CD19^+^ B lineage cells also co-expressed CD34 (Fig. 5h). These data demonstrate that ARID3a is required for B lineage maturation of young donor-derived HSCs in vitro. Myeloid versus lymphoid ratios in both the aged and young cultures with ARID3a modulation (Table 1) suggest that ARID3a is important for B lineage maturation in both aged and young HSCs. However, these data also suggest that aged HSCs may exhibit defects in function despite ARID3a expression.

**Table 1:** Modulation of ARID3a in HSCs alters Myeloid/Lymphoid ratios

	CD33+	CD19+	Myeloid/Lymphoid^a^
Aged - media	196 ± 16	108 ± 15	1.81
- control	262 ± 29	95 ± 26	2.76
- ARID3a	270 ± 39	738 ± 89	0.36
Young - media	216 ± 30	156 ± 19	1.38
- shCon	227 ± 30	228 ± 38	1.00
- shA3a	212 ± 26	52 ± 10	4.08

Average cell numbers ± SD for CD33 and CD19 expressing cells generated in mixed lineage differentiation cultures

^a^Myeloid/lymphoid ratio of CD33+ and CD19+ cells

**Fig. 6:**
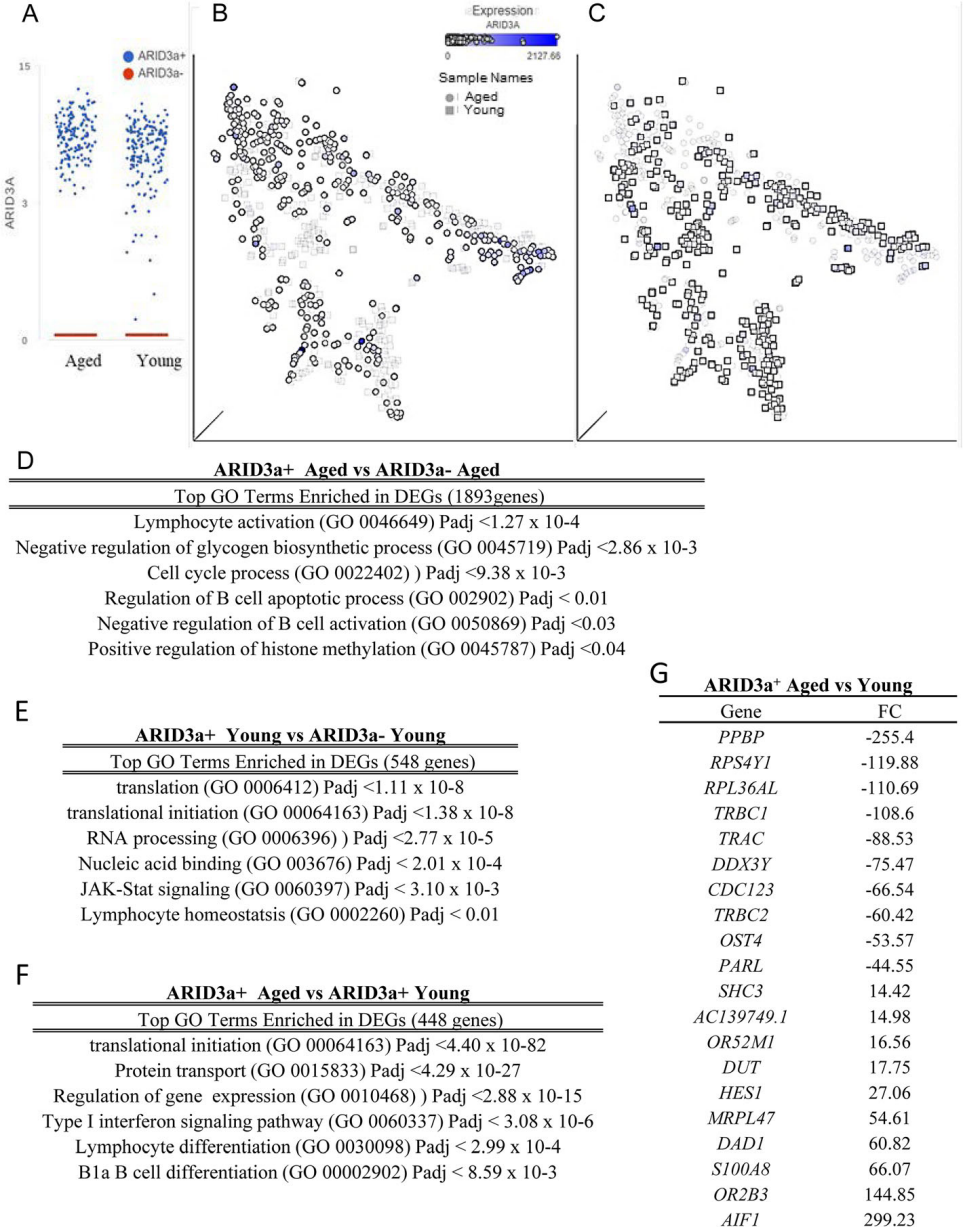
ARID3a^+^ HSCs from aged donors express altered transcriptomes compared to ARID3a^+^ HSCs from young donors. Single-cell RNA-seq expression profiles from 4 young (ages 19, 21, 37, and 40) and 4 aged (ages 61, 66, 68, and 70) donors were obtained and analyzed based on *ARID3A* transcript levels (*ARID3A*^*+*^ = > 0.5 CPM, *ARID3A*^*−*^ = < 0.5 CPM). Data from 264 young donor cells (172 *ARID3A*^*+*^ and 92 *ARID3A*^*−*^) and 301 aged donor cells (153 *ARID3A*^*+*^ and 148 *ARID3A*^*−*^) were assessed. **a** ARID3A transcript levels for young and aged donor cells are shown. tSNE plots of aged donor HSCs (circles) are overlaid with shaded young donor HSCs (squares) (**b**) and ARID3a expression levels are indicated by intensities of blue dots. In (**c**), tSNE plots are overlaid with young donor HSCs over the shaded aged donor HSCs for better visibility of the ARID3a-expressing cells in each group. The top GO terms enriched in DEGs from ARID3a^+^ vs ARID3a^−^ Aged HSCs are given with *p* values (**d**) and for young ARID3a^+^ versus ARID3a^−^ cells in (**e**). **f** Top GO terms directly comparing ARID3a^+^ cells in aged versus young donors are shown. **g** The most differentially expressed genes in ARID3a + HSCs in aged versus young donors are presented with negative or positive fold change (FC)

To identify genes associated with ARID3a expression in HSCs from aged versus young donors, we performed single cell RNA-seq analyses and analyzed each cell for the presence and level of *ARID3A* transcription as shown by the scatter plot (Fig. 6a). Analyses of *ARID3A* transcript by qPCR from bulk HSCs of known ARID3a protein expression suggest that transcript and protein expression in bulk HSCs correlate (data not shown). There were 153 ARID3a^+^ and 148 ARID3a^−^ cells from aged donors and 172 ARID3a^+^ and 92 ARID3a^−^ cells from the young donors. Three-dimensional t distributed stochastic neighbor embedding plots (tSNE) of 301 aged and 264 young HSCs from 8 donors revealed considerable spread in dimensionality in the aged (circles) versus young (squares), shown as overlays (Fig. 6b and c). This suggests that isolation of HSCs using standard surface markers (Fig. 1a) results in cells that are heterogeneous with respect to their transcriptomes in both aged and young donors. Identification of *ARID3A*-expressing cells, as indicated by blue symbols, reveals widespread *ARID3A* expression in both aged and young HSCs, with increased clustering of *ARID3A*-expressing cells toward the right-hand side in the aged donor cells (Fig. 6b). Ingenuity Pathway Analyses (IPA) analyses of *ARID3A*-associated genes and non-*ARID3A* associated genes from aged donors revealed enrichment in pathways associated with cell cycle, regulation of B cell apoptosis, negative regulation of B cell activation, and positive regulation of histone methylation in the *ARID3A* cells (Fig. 6d). Similar analyses of young donor cells indicated enrichment of pathways associated with lymphocyte homeostasis, JAK-STAT signaling and nucleic acid binding in the *ARID3A* cells (Fig. 6e).

Direct comparison of *ARID3A*-expressing cells from aged versus young donors revealed 253 genes down-regulated > 2-fold and 195 up-regulated genes. IPA of the *ARID3A*-associated genes from the young donors and the *ARID3A*-associated genes in the aged donors revealed enrichment in pathways associated with B1 B cell differentiation lymphocyte differentiation, type 1 interferon signaling, and regulation of gene expression in the aged donor cells (Fig. 6f). The most highly up-regulated gene in the aged *ARID3A*^+^ HSCs versus young donor *ARID3A*^+^ HSCS is *AIF1*, a gene associated with macrophage activation (Fig. 6g), while the most highly down-regulated gene encodes a chemokine, *PPBP*. In addition, at least four transcription factors *HES1, MAFB, ATF4* and *JAK1* are differentially regulated in aged versus young *ARID3A*-expressing HSCs. Thus, alterations in gene expression profiles in young versus aged *ARID3A*-expressing cells may be the result of changes in these regulatory proteins. Together, *ARID3A*-expressing cells in aged donors differ in gene expression patterns from *ARID3A*-expressing young donor HSCs.

## Discussion

The data presented here suggest that HSCs from both aged and young donors contain equivalent numbers of ARID3a-expressing cells, although total frequencies of ARID3a + cells were reduced in aged individuals. Analyses of hematopoietic lineage potential revealed that aged donor HSCs yielded reduced numbers of B cells compared to those derived from young donor HSCs, despite the presence of equivalent total numbers of ARID3a-expressing progenitors. These data suggest ARID3a expression alone is insufficient to cause age-associated shifts in B cell generation. Surprisingly, the aged donor-derived B cells retained CD34 expression to a greater degree than B lymphoid lineage cells derived from young donors, implying the aged donor B lineage cells could be less mature than young donor derived B cells. Artificial over-expression of ARID3a in aged donor-derived HSCs suggested that increased ARID3a expression resulted in better B lineage development without retention of surface CD34. ARID3a expression was critical for B lineage development from young donor peripheral blood-derived HSCs, consistent with our previous findings using HSCs from cord blood [30]. Importantly, single cell RNA-seq of both aged and young donor HSCs expressing *ARID3A* revealed a number of transcriptome differences between these cells, suggesting that the ARID3a-expressing cells in aged individuals differ at the molecular level from ARID3a-expressing cells from young individuals. These differences may contribute to the functional differences observed in hematopoiesis in vitro. Together, these data suggest that ARID3a-expressing progenitor cells in aged individuals differ at the transcription and functional level from those present in young individuals.

Previous studies on HSC-enriched HSPC populations from human report developmental skewing of aged cells toward myeloid lineage at the expense of lymphoid lineage cells [5, 7, 41]. While skewing toward the myeloid lineage in these in vitro cultures was minimal (Table 1), our studies differed from those previously published in several ways. We used isolated HSCs rather than the more heterogeneous HSPCs used by others. In addition, most other studies used myeloid colony forming assays in combination with liquid assays to enrich for myeloid development. Only one study used a liquid culture system to assess B cell and myeloid lineage development [7], though that study used a different feeder cell line and cytokine cocktail. Another possibility is that our aged precursors may have contained more pre-committed lineage progenitors for myeloid cells, as the frequencies of the ARID3a-expressing B cells were reduced in the aged HSCs. Thus, there may have been competition for B cell development due to pre-committed myeloid lineage cells. However, B lineage cytokines were present at equivalent levels in cultures of HSPCs from young donors that gave rise to more B lymphocytes. All of these factors are likely to contribute to the reduced myeloid/lymphoid ratio skewing we observed compared to previous reports.

We also observed increases in NK cells in the aged donor-derived cultures compared to the young donor-derived cells. Intriguingly, NK cells develop from precursors shared by B lymphocytes [42], and our unpublished results suggest that NK cells transcribe ARID3a. Others reported increased numbers of NK cells in the peripheral blood of aged individuals [43]. Further experiments would be merited to determine the potential relationship between B lineage and NK cell development in aged HSCs. Together, these data demonstrate that there are clear differences in the developmental potential of young and aged HSCs.

The aged donor B cells that developed in vitro retained CD34 expression, suggesting they may be less mature than the B lineage cells from young donor-derived cultures and highlighting at least one difference at the protein level between ARID3a-expressing cells from aged versus young donors. CD34 expression is reduced at the pre-B cell stage during normal B lymphopoiesis [44, 45]. Our previous data suggest that ARID3a is important for B lineage development at all early and immature stages of differentiation [30]. In this study, we could not meaningfully assess for which types of B lineage progenitors were present due to the limited numbers of cells generated in these assays. Irrespective of the developmental stage of B lineage cells derived from unmanipulated aged donor HSCs, when ARID3a expression was dramatically increased in those HSCs, they generated increased numbers of B lineage cells that no longer expressed CD34 and were presumably more mature. However, we cannot rule out the possibility that the cells that over-expressed ARID3a in those cultures were derived from some different subset of cells that did not have ARID3a originally. For example, myeloid progenitors, which are increased in old age, may have been diverted to the B lineage by over-expression of ARID3a (reviewed in [46]). Together, these data suggest that increased ARID3a expression in old age could improve B lineage development and perhaps allow increases in humoral immune responses in aged individuals.

In support of our surface expression data suggesting that the ARID3a-expressing cells from aged individuals differ from ARID3a + HSCs in the young, single cell transcriptome analyses revealed major differences in gene expression as well. GO analyses of young *ARID3A*-expressing HSCs and aged *ARID3A*-expressing HSCs reveal an enrichment in the pathways associated with B1 B cells differentiation in the aged cells (Fig. 6d). Several elegant studies from the Hardy group in the last few years have elucidated the role of ARID3a in B1 B cell development in fetal/neonatal murine development [21–23]. While human B1 cell identification remains controversial, others reported that cord blood HSPCs and mouse fetal liver HSPCs are very similar phenotypically and functionally [47, 48]. We previously found that ~ 75% of human umbilical cord blood HSCs express ARID3a where it is important for B lineage development [30]. These data suggest the interesting possibility that the ARID3a-expressing HSCs from aged donors could be enriched for B1 lineage-like development and might have fewer precursors that would ultimately result in conventional B lymphocytes. Murine bone marrow derived B1 B cells are suggested to be continually generated into old age [49], a population that has been reported to have separate progenitors than conventional B cells [41]. Alternatively, age-associated B cells (ABCs) are a unique heterogeneous subset of B cells that have been the focus of study for several laboratories in the last decade. First described separately by both the Cancro and Marrack research groups, these cells expand in old age and have been attributed to origins from multiple types of B lineage cells, including B1 B cells [50–52]. However, no one has demonstrated the origins of those cells from early hematopoietic progenitors. Our data suggest that alterations in humoral immunity attributed to alterations in B cell maturation in aged individuals are already evident at the gene level in early hematopoietic stem cells. The differences in gene expression that we observed between young and aged donor HSCs are generally consistent with published reports from others using young and aged HSPCs [35, 53]. However, as shown in Fig. 6, considerable heterogeneity remains at the transcriptome level even in purified HSCs. The ability to directly compare populations with the B cell-associated protein ARID3a underscores additional differences in aged versus young donor-derived HSCs, and present a new caveat that even within the ARID3a-expressing cells, some progenitors may be predisposed to develop into B1 versus conventional B cells as suggested by the GO analyses. More studies are clearly needed to better define the discrete progenitors within HSCs and the associated transcriptome differences between young and aged individuals.

## Conclusions

The data presented here demonstrate that ARID3a-expressing HSCs total numbers are equivalent between old and young donors, yet the aged donor HSCs produced fewer B cells in vitro. Functional defects in aged donor ARID3a-expressing HSCs were reflected both in expression of surface protein markers and at the transcriptome level, suggesting either that the ARID3a-expressing HSCs from aged donors are functionally defective in maturation responses, or that the HSCs expressing ARID3a in aged donors reflect skewing of a precursor subset of B lineage cells with different capacities to expand in these in vitro cultures. In addition, these data highlight the expansion of HSCs in aged individuals that do not express ARID3a, raising the possibility that these cells contribute to age-associated defects in hematopoiesis. Together, these data suggest that age-related defects in B cell function and humoral antibody responses may reflect changes that occur as early as the hematopoietic stem cell stage.

## Methods

### Cells

Peripheral blood mononuclear cells (PBMCs) from 54 healthy adults (ranging in age from 18 to 70) were isolated from fresh heparinized peripheral blood, or from leukocyte reduction chambers, with Ficoll-Pague Plus (GE Healthcare) or Lymphoprep (StemCell Technologies). Leukocyte reduction chambers typically contained 1–2 × 10^9^ PBMCs. Four million total PBMCs were stained for flow cytometric analyses and the remaining cells were either immediately enriched for CD34^+^ hematopoietic progenitors using the EasySep Human CD34 Positive Selection Kit (StemCell Technologies) or were cryopreserved for later enrichment. Thawed CD34^+^ enriched HSPCs were cultured overnight in Stem Pro media (Invitrogen) supplemented with 100 U penicillin and 100 μg streptomycin and a cytokine cocktail including stem cell factor (SCF) (100 ng/ml), FLT3 ligand (50 ng/ml), and thrombopoietin (TPO) (10 ng/ml) (R&D Systems) to acclimate cells prior to sorting. CD34-enriched cells were then sorted using a FACS Jazz (BD Biosciences) for CD38^−^ CD45RA^−^ CD49f^+^ HSCs for in vitro experiments [54]. Post-sort analyses were typically > 98% HSCs.

### Flow Cytometry

Cell surface markers were identified with the following fluorochromes: human hematopoietic lineage markers (CD2, CD3, CD14, CD16, CD19, CD56, CD235a) conjugated to APC (eBioscience), CD34 PE, CD38 Alexa Fluor 700, CD49f BV421, and CD45RA Brilliant Violet 570 (all from BioLegend). Appropriate isotype controls (BioLegend) were used for gating HSCs as described [29, 30]. In some cases, following surface marker staining, cells were fixed with fixation buffer (BD Biosciences), permeabilized with Transcription Factor Fixation/Permeabilization Buffer kit (eBioscience) and stained with goat anti-human ARID3a, followed by rabbit anti-goat FITC secondary antibody (Invitrogen) to identify ARID3a-expressing cells and/or with H2A.X phopspho (Ser139) APC-Fire750 (Biologend). Human anti-ARID3a peptide specific antibodies were generated and isolated over a peptide-specific column as described [55]. Cells were pretreated with anti-human CD32 antibody to block Fc receptor binding prior to surface staining to exclude non-specific binding. Doublet exclusion was used to ensure analyses of single cells prior to forward/side scatter gating. Data were collected using an LSRII (BD Biogenics) and FACSDiva (BD Biosciences) software version 4.1 or Stratedigm S1200Ex and CellCapTure acquisition software and were analyzed using FlowJo (Tree Star) software version 10.

### Telomere length analyses

Telomere lengths of sorted HSCs (5 young, 7 aged) were measured using the Relative Human Telomere Length Quantification qPCR assay kit (ScienCell Research Laboratories) according to the manufacturer’s protocol. Samples were chosen based on the trend line from estimated numbers of HSCs per mL of peripheral blood. Briefly, analyses were performed in duplicate for each sample, with control reference samples with predetermined telomere lengths provided by the manufacturer. Average telomere lengths were determined and the reference samples with a known telomere length was used to calculate the absolute telomere length of each individual HSC population.

### ARID3a modulation

FACS-purified HSCs (approximately 2000 cells per samples total divided between 3 treatments) were cultured in X-VIVO 10 medium (BioWhittaker) supplemented with 1% BSA, L-glutamine (Gibco), 100 U/mL penicillin-streptomycin (Invitrogen) and a cocktail of cytokines to allow hematopoietic lineage differentiation [36]: 100 ng/ml SCF (BioLegend), 100 ng/ml FLT3L, 15 ng/ml TPO, 10 ng/ml G-CSF (all R&D Systems) and 10 ng/ml IL-6 (Invitrogen) for transduction for 24 h in the presence of 4 μg/ml polybrene, as previously described [30]. Lentivirus containing shRNA specific for ARID3a or an unrelated scramble control shRNA with a co-expressed GFP reporter were prepared by Genecopoeia, Inc., as described previously [30]. Over-expression of full length human ARID3a was achieved using Lentiviral vectors or empty control vectors co-expressing an RFP reporter and prepared by ABM Inc. HSCs from young donors were treated with shRNA lentivirus using a multiplicity of infection (MOI) of 1. HSCs from aged donors were treated similarly with lentivirus expressing control and full-length ARID3a with RFP similarly, at an MOI of 1. Following transduction, HSCs were harvested, washed, counted and seeded at 100 cells per well in triplicate for each treatment (polybrene only, control vectors and experimental vectors) onto 1 × 10^4^ MS-5 murine stromal cells per well. Seeding numbers were chosen to ensure sufficient differentiation down multiple lineages and based on published data [56]. Stromal cells were plated 24 h previously in 96 well plates in H5100 medium (StemCell Technologies) supplemented with P/S, L-glutamine, and the following cytokines [36]: 100 ng/mL SCF, 10 ng/mL IL-2, 20 ng/mL IL-7 (BioLegend), 20 ng/mL IL-6 (Invitrogen), 10 ng/mL Flt3-L, 50 ng/mL TPO, 20 ng/mL G-CSF, and 20 ng/mL GM-CSF (R&D Systems) [36]. Cultures were fed by 50% volume change once a week. Cells were harvested after 3 weeks and assessed by flow cytometric analyses for myeloid lineage (CD33-PE-Cy5), NK lineage (CD56-BV421), and B lineage (CD19-BV650) cells. MS-5 cells were excluded from analyses using expression of mouse CD106-APC, as previously described [30]. Due to the small numbers of cells initially transfected, pre-culture assessments of transfection efficiencies could not be made at the initiation of culture, but were assessed at the conclusion of the cultures via flow cytometry for the presence of either GFP or RFP reporter expression, as these markers were co-expressed with the ARID3a shRNA or full length ARID3a, respectively.

### Single-cell RNA-seq

HSCs from 4 young (ages 19–40) and 4 aged (ages 61–70) individuals (2 males and 2 females each) were FACS-sorted and immediately used for single cell analyses. Smart-seq/C1 libraries were prepared on the OUHSC Consolidated Core Laboratory Fluidigm C1 system using the SMARTer Ultra Low RNA Kit (Clontech) according to the manufacturer’s protocol. Cells were loaded on a 5–10 μm RNA-seq microfluidic IFC at a concentration of 200,000/ml. Capture site occupancy was surveyed using a standard light microscope and recorded to verify cell capture. Sequencing library amplification was performed using Nextera XT Index primers (Illumina) according to manufacturer’s protocol. Barcoded library concentration and fragment size distribution was determined using Agilent High Sensitivity D1000 kit on an Agilent 2200 TapeStation (Agilent Technologies) at the OMRF Genomics Core Facility. Paired-end (2 × 50 bp) sequencing was performed on a NovaSeq 6000 platform by the OMRF Genomics Core Facility.

Paired-end reads were aligned to the hg38 genome assembly with STAR using default parameters. All sequencing was performed on the same day to minimize batch effects from individual donors and they were further reduced using ComBat (Johnson et al., Biostatistic vol 8 issue 1, pgs 118–127, 2007) within the Partek Genomics suite on the gene expression matrices for young and old donors. The Partek Genomics Suite was used to process the aligned reads to estimate gene expression levels. Cells that had greater than 25% of counts mapping to mitochondrial genes indicate stressed or dying cells and were excluded from analyses. The number of detected genes was 44,025 across 301 cells for aged samples and 39,417 genes across 264 cells for young samples. Log normalized counts per millions (CPM) were log_2_-transformed and used for tSNE visualization and differential expression analyses. Data from aged and young samples were interrogated separately based on ARID3a expression, with > 0.5 CPM denoting *ARID3A*^*+*^ cells and < 0.5 CPM denoting *ARID3A*^−^ cells for further analysis. ANOVA analysis was performed within the Partek Flow software to identify DEGs with 2-fold or greater changes in expression and a False Discovery Rate (FDR) < 0.05. IPA analyses were performed on significant DEGs. RNA-seq data are publicly available through the GEO NCBI database under the accession number GSE138544.

### Data analyses

Data were statistically evaluated using the non-parametric Mann Whitney U test or the non-parametric Wilcoxon paired test to compare distribution of variables between groups. Correlations were evaluated using Pearson’s Correlations. Statistical analysis was performed with Prism (Graphpad) software version 8.2. Differential gene expression was analyzed using ANOVA within the Partek Genomics Suite. Gene ontology analyses on differentially expressed genes were analyzed within the Partek Flow Genomics Suite using the gene set enrichment tool. *P* values of less than 0.05 were considered significant.

## Data Availability

The RNA-seq data that support the findings of this study are openly available through the GEO NCBI database under the accession number listed in the methods section upon publication (GSE138544). Reviewers will be given coded access to these metadata sets upon request by the editor. All other supporting data for the finding of these studies are available from the corresponding author upon request.

## References

[1] Colby SL, Ortman JM (2015). Projections of the Size and Composition of the U.S. Population: 2014 to 2060. Population Estimates and Projections. Current Population Reports. P25–1143. Numerical/Quantitative Data Report.

[2] Pang WW, Schrier SL, Weissman IL (2017). Age-associated changes in human hematopoietic stem cells. Semin Hematol.

[3] Laurenti E, Gottgens B (2018). From haematopoietic stem cells to complex differentiation landscapes. Nature..

[4] Dykstra B, Olthof S, Schreuder J, Ritsema M, de Haan G (2011). Clonal analysis reveals multiple functional defects of aged murine hematopoietic stem cells. J Exp Med.

[5] Kuranda K, Vargaftig J, de la Rochere P, Dosquet C, Charron D, Bardin F (2011). Age-related changes in human hematopoietic stem/progenitor cells. Aging Cell.

[6] Pang WW, Price EA, Sahoo D, Beerman I, Maloney WJ, Rossi DJ (2011). Human bone marrow hematopoietic stem cells are increased in frequency and myeloid-biased with age. Proc Natl Acad Sci U S A.

[7] Sun D, Luo M, Jeong M, Rodriguez B, Xia Z, Hannah R (2014). Epigenomic profiling of young and aged HSCs reveals concerted changes during aging that reinforce self-renewal. Cell Stem Cell.

[8] Fali T, Fabre-Mersseman V, Yamamoto T, Bayard C, Papagno L, Fastenackels S (2018). Elderly human hematopoietic progenitor cells express cellular senescence markers and are more susceptible to pyroptosis. JCI Insight.

[9] Flach J, Bakker ST, Mohrin M, Conroy PC, Pietras EM, Reynaud D (2014). Replication stress is a potent driver of functional decline in ageing haematopoietic stem cells. Nature..

[10] Kortschak RD, Tucker PW, Saint R (2000). ARID proteins come in from the desert. Trends Biochem Sci.

[11] Patsialou A, Wilsker D, Moran E (2005). DNA-binding properties of ARID family proteins. Nucleic Acids Res.

[12] Ratliff ML, Templeton TD, Ward JM, Webb CF (2014). The bright side of hematopoiesis: regulatory roles of ARID3a/bright in human and mouse hematopoiesis. Front Immunol.

[13] Popowski M, Templeton TD, Lee BK, Rhee C, Li H, Miner C (2014). Bright/Arid3A acts as a barrier to somatic cell reprogramming through direct regulation of Oct4, Sox2, and Nanog. Stem Cell Rep.

[14] Rajaiya J, Nixon JC, Ayers N, Desgranges ZP, Roy AL, Webb CF (2006). Induction of immunoglobulin heavy-chain transcription through the transcription factor bright requires TFII-I. Mol Cell Biol.

[15] Rajaiya J, Hatfield M, Nixon JC, Rawlings DJ, Webb CF (2005). Bruton’s tyrosine kinase regulates immunoglobulin promoter activation in association with the transcription factor bright. Mol Cell Biol.

[16] Lin D, Ippolito GC, Zong RT, Bryant J, Koslovsky J, Tucker P (2007). Bright/ARID3A contributes to chromatin accessibility of the immunoglobulin heavy chain enhancer. Mol Cancer.

[17] Ward JM, Ratliff ML, Dozmorov MG, Wiley G, Guthridge JM, Gaffney PM (2016). Expression and methylation data from SLE patient and healthy control blood samples subdivided with respect to ARID3a levels. Data Brief.

[18] Ward JM, Ratliff ML, Dozmorov MG, Wiley G, Guthridge JM, Gaffney PM (2016). Human effector B lymphocytes express ARID3a and secrete interferon alpha. J Autoimmun.

[19] Webb CF, Bryant J, Popowski M, Allred L, Kim D, Harriss J (2011). The ARID family transcription factor bright is required for both hematopoietic stem cell and B lineage development. Mol Cell Biol.

[20] Nixon JC, Ferrell S, Miner C, Oldham AL, Hochgeschwender U, Webb CF (2008). Transgenic mice expressing dominant-negative bright exhibit defects in B1 B cells. J Immunol.

[21] Hayakawa K, Li YS, Shinton SA, Bandi SR, Formica AM, Brill-Dashoff J (2019). Crucial role of increased Arid3a at the pre-B and immature B cell stages for B1a cell generation. Front Immunol.

[22] Li YS, Zhou Y, Tang L, Shinton SA, Hayakawa K, Hardy RR (2015). A developmental switch between fetal and adult B lymphopoiesis. Ann N Y Acad Sci.

[23] Zhou Y, Li YS, Bandi SR, Tang L, Shinton SA, Hayakawa K (2015). Lin28b promotes fetal B lymphopoiesis through the transcription factor Arid3a. J Exp Med.

[24] Simell B, Vuorela A, Ekstrom N, Palmu A, Reunanen A, Meri S (2011). Aging reduces the functionality of anti-pneumococcal antibodies and the killing of Streptococcus pneumoniae by neutrophil phagocytosis. Vaccine..

[25] Brooks LRK, Mias GI (2018). Streptococcus pneumoniae’s virulence and host immunity: aging, diagnostics, and prevention. Front Immunol.

[26] Oldham AL, Miner CA, Wang HC, Webb CF (2011). The transcription factor bright plays a role in marginal zone B lymphocyte development and autoantibody production. Mol Immunol.

[27] Baumgarth N (2017). A hard(y) look at B-1 cell development and function. J Immunol.

[28] Sanz I, Wei C, Jenks SA, Cashman KS, Tipton C, Woodruff MC (2019). Challenges and opportunities for consistent classification of human B cell and plasma cell populations. Front Immunol.

[29] Ratliff ML, Ward JM, Merrill JT, James JA, Webb CF (2015). Differential expression of the transcription factor ARID3a in lupus patient hematopoietic progenitor cells. J Immunol.

[30] Ratliff ML, Mishra M, Frank MB, Guthridge JM, Webb CF (2016). The transcription factor ARID3a is important for in vitro differentiation of human hematopoietic progenitors. J Immunol.

[31] Rossi DJ, Bryder D, Zahn JM, Ahlenius H, Sonu R, Wagers AJ (2005). Cell intrinsic alterations underlie hematopoietic stem cell aging. Proc Natl Acad Sci U S A.

[32] Wahlestedt M, Norddahl GL, Sten G, Ugale A, Frisk MA, Mattsson R (2013). An epigenetic component of hematopoietic stem cell aging amenable to reprogramming into a young state. Blood.

[33] Zhang WG, Zhu SY, Bai XJ, Zhao DL, Jian SM, Li J (2014). Select aging biomarkers based on telomere length and chronological age to build a biological age equation. Age (Dordr).

[34] Lee Y, Sun D, Ori APS, Lu AT, Seeboth A, Harris SE (2019). Epigenome-wide association study of leukocyte telomere length. Aging (Albany NY).

[35] de Haan G, Lazare SS (2018). Aging of hematopoietic stem cells. Blood..

[36] van Galen P, Kreso A, Wienholds E, Laurenti E, Eppert K, Lechman ER (2014). Reduced lymphoid lineage priming promotes human hematopoietic stem cell expansion. Cell Stem Cell.

[37] Camous X, Pera A, Solana R, Larbi A (2012). NK cells in healthy aging and age-associated diseases. J Biomed Biotechnol.

[38] Cichocki F, Grzywacz B, Miller JS (2019). Human NK cell development: one road or many?. Front Immunol.

[39] Sanz E, Munoz AN, Monserrat J, Van-Den-Rym A, Escoll P, Ranz I (2010). Ordering human CD34+CD10-CD19+ pre/pro-B-cell and CD19- common lymphoid progenitor stages in two pro-B-cell development pathways. Proc Natl Acad Sci U S A.

[40] Dmytrus J, Matthes-Martin S, Pichler H, Worel N, Geyeregger R, Frank N (2016). Multi-color immune-phenotyping of CD34 subsets reveals unexpected differences between various stem cell sources. Bone Marrow Transplant.

[41] Montecino-Rodriguez E, Dorshkind K (2012). B-1 B cell development in the fetus and adult. Immunity..

[42] Doulatov S, Notta F, Laurenti E, Dick JE (2012). Hematopoiesis: a human perspective. Cell Stem Cell.

[43] Przemska-Kosicka A, Childs CE, Maidens C, Dong H, Todd S, Gosney MA (2018). Age-related changes in the natural killer cell response to seasonal influenza vaccination are not influenced by a Synbiotic: a randomised controlled trial. Front Immunol.

[44] Ichii M, Oritani K, Yokota T, Schultz DC, Holter JL, Kanakura Y (2010). Stromal cell-free conditions favorable for human B lymphopoiesis in culture. J Immunol Methods.

[45] Ichii M, Oritani K, Yokota T, Zhang Q, Garrett KP, Kanakura Y (2010). The density of CD10 corresponds to commitment and progression in the human B lymphoid lineage. PLoS One.

[46] Mejia-Ramirez E, Florian MC (2020). Understanding intrinsic hematopoietic stem cell aging. Haematologica..

[47] Lepus CM, Gibson TF, Gerber SA, Kawikova I, Szczepanik M, Hossain J (2009). Comparison of human fetal liver, umbilical cord blood, and adult blood hematopoietic stem cell engraftment in NOD-scid/gammac−/−, Balb/c-Rag1−/−gammac−/−, and C.B-17-scid/bg immunodeficient mice. Hum Immunol.

[48] Harrison DE, Astle CM (1997). Short- and long-term multilineage repopulating hematopoietic stem cells in late fetal and newborn mice: models for human umbilical cord blood. Blood..

[49] Holodick NE, Rothstein TL (2015). B cells in the aging immune system: time to consider B-1 cells. Ann N Y Acad Sci.

[50] Cancro MP (2020). Age-associated B Cells. Annu Rev Immunol.

[51] Hao Y, O'Neill P, Naradikian MS, Scholz JL, Cancro MP (2011). A B-cell subset uniquely responsive to innate stimuli accumulates in aged mice. Blood..

[52] Rubtsov AV, Rubtsova K, Fischer A, Meehan RT, Gillis JZ, Kappler JW (2011). Toll-like receptor 7 (TLR7)-driven accumulation of a novel CD11c(+) B-cell population is important for the development of autoimmunity. Blood..

[53] Rundberg Nilsson A, Soneji S, Adolfsson S, Bryder D, Pronk CJ (2016). Human and murine hematopoietic stem cell aging is associated with functional impairments and intrinsic megakaryocytic/Erythroid bias. PLoS One.

[54] Notta F, Doulatov S, Laurenti E, Poeppl A, Jurisica I, Dick JE (2011). Isolation of single human hematopoietic stem cells capable of long-term multilineage engraftment. Science (New York, NY).

[55] Nixon JC, Rajaiya JB, Ayers N, Evetts S, Webb CF (2004). The transcription factor, bright, is not expressed in all human B lymphocyte subpopulations. Cell Immunol.

[56] Tomellini E, Fares I, Lehnertz B, Chagraoui J, Mayotte N, MacRae T (2019). Integrin-alpha3 Is a Functional Marker of Ex Vivo Expanded Human Long-Term Hematopoietic Stem Cells. Cell Rep.

